# Brainiac Caspases: Beyond the Wall of Apoptosis

**DOI:** 10.3389/fncel.2019.00500

**Published:** 2019-11-05

**Authors:** Ana María Espinosa-Oliva, Juan García-Revilla, Isabel María Alonso-Bellido, Miguel Angel Burguillos

**Affiliations:** Departamento de Bioquímica y Biología Molecular, Facultad de Farmacia, Universidad de Sevilla, and Instituto de Biomedicina de Sevilla (IBiS), Hospital Universitario Virgen del Rocío/CSIC, Seville, Spain

**Keywords:** caspase, neuron, astrocytes, oligodendrocytes, inflammasome, necroptosis, neurodegeneration, pyroptosis

## Abstract

For the last two decades, caspases, a family of cysteine-aspartic proteases, have evolved from being considered solely as regulators of apoptosis or inflammation to having a wider range of functions. In this mini review, we focus on the most recent “non-apoptotic” roles of caspases in the CNS, particularly in neurons, astrocytes and oligodendrocytes. Non-apoptotic caspase functions in microglia have already been reviewed extensively elsewhere. Here we discuss the involvement of caspases in the activation of the inflammasome, autophagy, and non-apoptotic forms of cell death such as necroptosis and pyroptosis. Also, we review the involvement of caspases in synapses and the processing of aggregates key to neurodegenerative diseases such as Parkinson’s, Alzheimer’s and Huntington’s diseases. Likewise, we mention the recently described involvement of caspases in mitochondrial biogenesis, which is a function independent of the enzymatic activity. We conclude discussing the relevance that “new” functions of caspases have in the CNS and the future of this field of research.

## Introduction

Caspases are a family of proteins belonging to the cysteine aspartate proteases classically associated with different forms of programmed cell death (Stennicke and Salvesen, 1999; Hyman and Yuan, 2012; Tummers and Green, 2017).

Since the discovery of CED-3 in *Caenorhabditis elegans* (Ellis and Horvitz, 1986) and the caspase-1/Interleukin-1 converting enzyme (ICE) (Thornberry et al., 1992; Yuan et al., 1993), caspases were grouped based on their function as “apoptotic” or “inflammatory.” This classification has remained useful to some extent until recently, since new non-apoptotic or non-inflammatory roles have surfaced for caspases (Shalini et al., 2015; Baena-Lopez et al., 2018; Hollville and Deshmukh, 2018). Besides, over the last decade, evidence has been gathered detailing non-apoptotic roles for caspases in astrocytes, neurons, oligodendrocytes (ODCs) and microglia (Acarin et al., 2007; Li et al., 2010; Burguillos et al., 2011; Wagner et al., 2011).

The aim of this mini-review is to provide an update on the various functions of caspases (from those described over two decades ago, to recent functions described within the last 5 years) in the CNS, focusing mainly on neurons, astrocytes and ODCs (see Table 1). We will only briefly discuss microglia cells as we recently published an in-depth review on this topic (Shen et al., 2018).

**TABLE 1:** Non-apoptotic functions of caspases in different cell types.

	**Cell type**	**Model and caspase involved**	**References**
**Inflammasome**	**Astrocytes**	Primary cortical astrocytes from mice with Aβ_1__–__42_ (**caspase-1**)	Ebrahimi et al., 2018
		Primary glial cultures from WT, Nlrc4^–/–^, Nlrp3^–/–^ and Asc^–/–^ mice with LPC(**caspase-1**)	Freeman et al., 2017;
		Human primary astrocytes with ATP (**caspase-1**)	Minkiewicz et al., 2013
		SOD1 mouse model/ALS patients (**caspase-1**)	Johann et al., 2015
		Intracerebral hemorrhage mouse model (**caspase-1**)	Wang et al., 2017
		Mouse primary astrocytes with methamphetamine (**caspase-11**)	Du et al., 2017
	**Microglia**	EAE mouse model (**caspase-8**)	Zhang et al., 2018
	**Neurons**	Primary cultures of human neurons under serum-deprived conditions (**caspase-1**)	Kaushal et al., 2015
		APPSwe/PS1dE9 transgenic mice (**caspase-1**)	Tan et al., 2014
		Dopaminergic neurons from PD patients (**caspase-1**)	von Herrmann et al., 2018
	**ODCs**	Administration prenatal of dexamethasone to mice (**caspase-1**)	Maturana et al., 2017
**Pyroptosis**	**Astrocytes**	Ischemia induced by the oxygen-glucose deprivation in primary cultured astrocytes from rats (**caspase-1**)	Xia et al., 2018
		Stroke model in rats (**caspase-1**)	
		Cultured rat cortical astrocytes with bilirubin (**caspase-1**)	Feng et al., 2018
		Sepsis model in mice and rats induced by LPS (**caspase-1**)	Li et al., 2019; Sun et al., 2019
	**Neurons Neurons**	Primary cultures of human neurons under serum-deprived conditions (**caspase-1**)	Kaushal et al., 2015
		Cultured cortical neurons from rats with Aβ (**caspase-1**)	Tan et al., 2014
		SCI model in rats (**caspase-1, caspase-11**)	Lin et al., 2016; de Rivero Vaccari et al., 2008
		Caspase-1-/- mice subjected to controlled cortical impact injury (**caspase-1**)	Liu et al., 2018
		Ischemic stroke model in mice (**caspase-1**, **caspase-11**)	Fann et al., 2018
		Ocular hypertension-injured retina in mice (**caspase-1**)	Pronin et al., 2019
	**ODCs**	EAE mouse model (**caspase-1**)	McKenzie et al., 2018
		MS patients (**caspase-1**)	
**Necroptosis**	**ODCs**	EAE and Cuprizone mouse models of MS and in MS patients (**caspase-8**)	Ofengeim et al., 2015
		Osmotic demyelination syndrome model in mice (**caspase-8**)	Bouchat et al., 2018
		Neonatal rats subjected to hypoxia-ischemia (**caspase-8**)	Qu et al., 2017
	**Neurons**	Global cerebral ischemia/reperfusion in CA1 neurons in rats (**caspase-8**)	Xu et al., 2016
		Subarachnoid hemorrhage induced brain injury model in rats (**caspase-8**)	Chen et al., 2018; Yuan et al., 2019
		Models of retinal degeneration in rats (**caspase-8**)	Jang et al., 2019
	**Astrocytes**	Mouse spinal cord astrocytes with LPS or TNF-α with zVAD (**caspase-8**)	Fan et al., 2016
		SCI model in mice (**caspase-8**)	
	**Microglia**	Mice stereotaxically injected with LPC (**caspase-8**)	Lloyd et al., 2019
**Synapses**	**Neurons**	Cultured neurons subjected to neurotrophic factor deprivation (**caspase-3**, **caspase-6**, **caspase-9**)	Simon et al., 2012
		Caspase-3 knockout mice (**caspase-3**)	Ertürk et al., 2014
		J20 APP transgenic mice (**caspase-2**)	Pozueta et al., 2013
		Caspase-9^–/–^ mice (**caspase-9**)	Ohsawa et al., 2010
		Caspase-3^–/–^neonatal mice (**caspase-3**)	Gu et al., 2017
		Caspase-3 knockout mice and rats (**caspase-3**)	Li et al., 2010
		Tg2576-APPSwe mice (**caspase-3**)	D’Amelio et al., 2011
**Autophagy**	**Neurons**	Primary cortical neurons from wt and casp2^–/–^ mice with rotenone (**caspase-2**)	Tiwari et al., 2011
		PC12 cells and cortical neurons from rats with Aβ_1__–__42_ (**caspase-3**)	Wang et al., 2017
		APPSwe/PS1dE9 transgenic mice (**caspase-3**)	
**Mitochondria Biogenesis**	**Neurons**	Dopaminergic cell line (**procaspase-3**)	Kim et al., 2018

### Caspases Inflammasome and Pyroptosis

**FIGURE 1:**
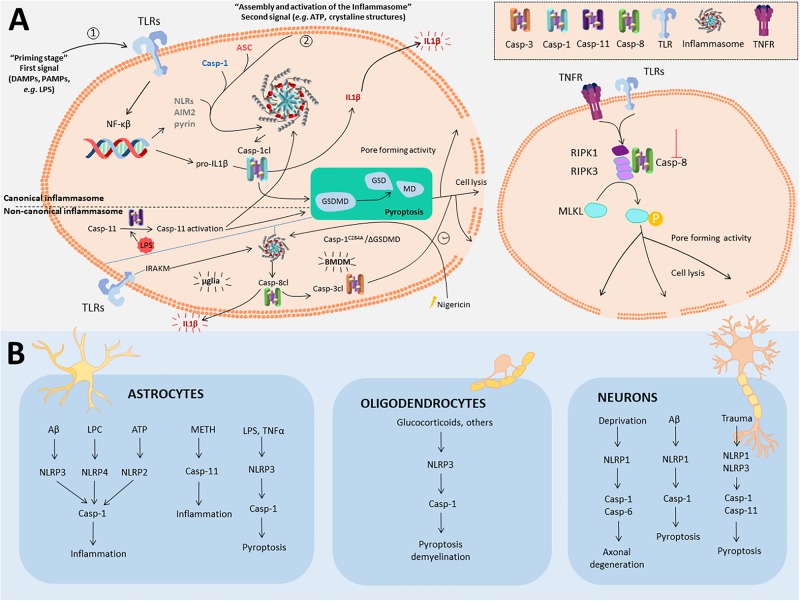
Non-apoptotic roles for caspases in inflammasome, pyroptosis and necroptosis in the CNS. **(A)** Left panel represents the general mechanisms to trigger both canonical and non-canonical inflammasome activation and subsequent pyroptosis. Also, it is included the recently described caspase-8 dependent non-canonical inflammasome activation in microglia (mglia) and BMDM. Canonical inflammasome commences upon a first signal (“priming stage,” 1) induced by DAMPS or PAMPS that promote the expression of the components of the inflammasome. Three types of inflammasome have been described so far (NLRs, AIM2, and Pyrin inflammasomes). A second signal (2) is needed for inflammasome assembly via the protein adaptor ASC and caspase-1, which becomes active through cleavage. Cleaved caspase-1 (Casp-1cl) may either cleave pro-IL1β into IL1β that will be released, or process gasdermin D (GSDMD), the known effector of pyroptosis. In the non-canonical inflammasome, cytosolic LPS, from intracellular Gram negative bacteria binds with high affinity to caspase-11, provoking self-assembly and its activation. Active caspase-11 indirectly promotes the cleavage of the pro-IL1β by activating the NLRP3 inflammasome and caspase-1. Moreover, caspase-11 is also capable to directly cleave GSDMD to promote pyroptosis. Caspase-8 gets activated via the inflammasome in two different ways depending on the cell type. In microglia, the inflammasome gets activated via TLR-IRAKM axis, that promotes caspase-8 activation and IL-1β release. In BMDM, in the absence of GSDMD or caspase-1 activity, the inflammasome becomes activated upon Nigericin treatment, also promoting caspase-8 activation and inducing IL1β release and delayed cell lysis, via caspase-3, and independent of GSDMD. Necroptosis (right panel) is a programmed form of necrosis commonly triggered by tumor necrosis factor receptor (TNFR) or toll-like receptors (TLRs) upon inflammatory or neurodegenerative stimuli. Activation of these receptors leads to receptor-interacting serine/threonine-protein kinase 1 (RIPK1) disengagement from the receptor platform and the rapid recruitment of RIPK3 and Casp8 to form the ripoptosome. At this point, activation of caspase-8 will lead to apoptosis. However, absence or inhibition of caspase-8 will lead to phosphorylation of both RIPK1 and RIPK3 and the recruitment and phosphorylation of mixed lineage kinase domain like pseudokinase (MLKL). MLKL phosphorylation triggers its oligomerization, gaining pore-forming activity that leads to cell lysis. **(B)** Inflammasomes described in different CNS cell types described in this paper.

The inflammasome (Martinon et al., 2002) is a multiprotein intracellular complex that senses pathogenic microorganisms and sterile stressors, which ultimately processes and releases IL-1β and IL-18. The activation of the inflammasome consists of a two-step pathway (First step: TLR activation; Second step: e.g., ATP). This leads to caspase-1, caspase-11 (de Rivero Vaccari et al., 2014; Walsh et al., 2014; Broz and Dixit, 2016; Voet et al., 2019) and caspase-8 (Zhang et al., 2018) activation. Also intracellular lipopolysaccharide (LPS) may bind directly to caspase-11, promoting its activation (Shen et al., 2018). Inflammasomes are categorized as canonical (caspase-1) or non-canonical (caspase-11) (Broz and Dixit, 2016) (for the general signaling pathway, see Figure 1A). In microglia cells, a recent study described a non-canonical inflammasome associated to caspase-8 (and independently of caspase-1), in which microglia were able to generate IL-1β in an Experimental autoimmune encephalomyelitis (EAE) mouse model (Zhang et al., 2018). This finding is contradictory of a previous study done in Bone Marrow Derived Macrophages (BMDM), where caspase-1 deficiency is necessary for caspase-8 processing and release of IL-1β (Schneider et al., 2017).

The activation of the inflammasome can evolve into a process of regulated cell death termed pyroptosis, which acts as a defense mechanism against infection by inducing pathological inflammation (Tait et al., 2014). In pyroptosis, both caspase-1 and caspase-11 (caspase-4 and caspase-5 in humans) have been described as mediators of this type of cell death (for the general signaling pathway, see Figure 1A; Aglietti et al., 2016; Pronin et al., 2019).

Inflammasomes were originally described in immune cells (Shen et al., 2018) though the presence of the inflammasome in other cells than microglia in the CNS has been reported too. In astrocytes, crucial regulators of the immune responses in the injured CNS, the presence of canonical and non-canonical inflammasomes occurs upon treatment with different stimuli *in vitro* (Minkiewicz et al., 2013; Du et al., 2017; Freeman et al., 2017; Ebrahimi et al., 2018). *In vivo*, high expression levels of nucleotide-binding oligomerization domain-like receptor protein (NLRP) 3 related to the neuroinflammatory response has been found in astrocytes of SOD1 mice and in Amyotrophic lateral sclerosis (ALS) patients (Johann et al., 2015). Also, different studies showed astrocytic pyroptosis in animal models of sepsis induced by LPS (Li et al., 2019; Sun et al., 2019).

Besides microglia and astrocytes, the presence of different types of inflammasomes have been reported in non-immune related cells, such as neurons (Figure 1B and Table 1). Over 10 years ago, the existence of NACHT leucine-rich-repeat protein 1 (NALP1) neuronal inflammasome was reported (Kummer et al., 2007). Since then, several studies have demonstrated that activation of the inflammasome occurs in neurons, followed normally by cell death induced by pyroptosis. In the context of Alzheimer’s disease (AD**),** pyroptosis occurs upon amyloid-β (Aβ) treatment *in vitro*, as well as *in vivo* in the APPSwe/PS1dE9 mice. In both cases, either knockdown of NLRP1 (another type of inflammasome) or caspase-1 conferred neuroprotection (Tan et al., 2014). Interestingly, in a recent study, NLRP3 inflammasome was characterized in dopaminergic neurons from Parkinson’s disease (PD) patients (von Herrmann et al., 2018). In this study the authors identified, via exome sequencing, a single-nucleotide polymorphisms (SNPs) for NLRP3 which results in a less soluble form of NLRP3 protein than NLRP3 wild-type. This less soluble form was associated with a significantly reduced risk of developing PD, highlighting the relevance of NLRP3 inflammasome in dopaminergic neurons in PD (von Herrmann et al., 2018).

In a recent study by Tsuchiya et al. (2019), the authors described how it was possible to alter the type of neuronal death (from pyroptosis to apoptosis), based on the levels of expression of Gasdermin D (GSDMD). GSDMD is a specific substrate for caspase-1, -4, -5 and -11, whose N-terminal cleavage product has a pore-forming activity that causes cell swelling and lysis via pyroptosis. In this sense, cortical neurons may die through apoptosis instead of pyroptosis after oxygen-glucose deprivation (OGD) and nigericin stimulation due to low expression of GSDMD (Tsuchiya et al., 2019).

Finally, in ODCs, the relevance of inflammasome activation has not been as well documented, with only a few reports published. For instance, prenatal administration of dexamethasone to mice, a treatment that promotes demyelination, induces NLRP3, caspase-1 and Apoptosis-associated speck-like protein containing a CARD (ASC) expression in ODCs, which the authors suggested could be contributing to demyelination (Maturana et al., 2017). Also, pyroptosis has been observed in myelin-forming ODCs in the EAE mice and in patients with Multiple sclerosis (MS) (McKenzie et al., 2018). In this case, inhibition of caspase-1, using the inhibitor VX-756, reduced pyroptosis in ODCs and microglia and promoted neuroprotection and improved performance in different neurobehavioral tests. In this case is hard to distinguish if VX-756 beneficial effect over behavior is based on inhibition of inflammasome and/or pyroptosis or both.

### Caspases and Necroptosis

Originally necroptosis was considered a passive death of cells under pathological conditions, where the cellular contents are released and cause an immune response. However, currently it is considered a cell death pathway regulated through the interaction of different molecules including receptor-interacting protein kinase (RIPK)-1 and RIPK3, mixed lineage kinase domain-like protein (MLKL), and caspase-8 (whose activity is inhibited for instance by caspase-8 or pan-caspase inhibitors) (Figure 1A; Tait et al., 2014; Ofengeim et al., 2015).

Necroptosis mediates ODC degeneration induced by TNF-α, whose binding to TNFR1 triggers caspase-8 activation, and the inhibition of RIPK1 protects against ODC cell death in two animal models to study MS, and also in cell culture (Ofengeim et al., 2015). In the same paper, the authors found in cortical lesions of human MS samples defective caspase-8 activation and activation of RIPK1, RIPK3, and MLKL. Also in a different *in vivo* model of osmotic demyelination syndrome, ODC cell death has been linked to necroptosis based on an increase of phospho-MLKL immunoreactivity (Bouchat et al., 2018), but further experiments are needed to confirm this result.

In the developing brain, a study showed that neonatal rats subjected to hypoxia–ischemia (HI) and ODCs from neonatal rats treated with OGD together with the pan-caspase activity inhibitor zVAD die via necroptosis (Qu et al., 2017). Treatments aimed to block the interaction of RIPK3 with MLKL or CaMKIIδ, a new described substrate of RIPK3, managed to decrease necroptosis under these conditions (Qu et al., 2017).

Interestingly in neurons, a new interaction of RIPK3 with the protein apoptosis-inducing factor (AIF) has been reported. Their interaction and translocation into the nucleus in a model of 20-min global cerebral ischemia/reperfusion (I/R) has been described in CA1 neurons, which was critical to ischemic DNA degradation and programmed necrosis (Xu et al., 2016). The fact that these CA1 neurons lack caspase-8 expression, facilitates necroptosis in these cells.

In astrocytes, a study performed in mouse spinal cord astrocytes treated with LPS or TNF-α with zVAD *in vitro*, and subjected to spinal cord injury (SCI) *in vivo*, showed an increase in the expression of RIPK3 and MLKL proteins (Fan et al., 2016). Treatment with the chemical inhibitor of RIPK1 (Necrostatin-1) or RIPK3 genetic ablation (Fan et al., 2016) rescued the cells.

In the case of neurons, in a model of subarachnoid hemorrhage it has been reported that neuronal death occurs via RIPK3 and MKLK (Chen et al., 2018; Yuan et al., 2019). Moreover, inhibition of RIPK1 [using RIPK1-inhibitory compound (RIC)] in retinal degenerative diseases induced by glaucomatous insult has been proved to exert a neuroprotective effect (Jang et al., 2019).

Intriguingly, while necroptosis of neurons, ODCs and astrocytes is associated normally with demyelination and neurodegeneration, a new report in microglia cells (Lloyd et al., 2019) proposes that the remyelination process in white matter is driven by proinflammatory microglia necroptosis and the subsequent repopulation of positively regulated by type-1 IFN signaling microglia.

### Caspases and Synapses

In the mature CNS, caspases are not only involved in mediating cell death but also regulatory events that are important for neuronal functions, such as, axon pruning and synapse elimination (D’Amelio et al., 2010; Hyman and Yuan, 2012; Hollville and Deshmukh, 2018).

During axonal pruning, the absence of neurotrophic factors provokes caspase-3/6 activation via JNK that facilitates mitochondrial depolarization (Hollville and Deshmukh, 2018). The role of caspases in axon pruning has also been studied in the context of axon degeneration induced by neurotrophic factor deprivation in sympathetic neurons. Caspase-3, caspase-6 or caspase-9 are required for the axon selective degeneration or pruning (Simon et al., 2012) but not Apaf-1 (Cusack et al., 2013), which is required for triggering cell death upon axonal Neuronal Growth Factor (NGF) deprivation. Caspase-3 was also found to control spine density and dendrite morphology in specific areas within the cell generating supernumerary spines in caspase-3 knockout mice (Ertürk et al., 2014). Caspase-2 participates in the control of dendrite spine density via activation of the RhoA/ROCK-II signaling pathway (Pozueta et al., 2013). Caspase-2 deficiency in J20 APP transgenic mice did not thwart cognitive function despite having similar Aβ load and neuroinflammatory response to wild type animals (Pozueta et al., 2013).

Caspases are also involved in axonal guidance and synaptogenesis like for instance caspase-9-mediated cleavage of Semaphorin 7 mediates the proper projection of axons in sensory neurons (Ohsawa et al., 2010). In the spinal cord of neonatal mice, caspase-3 regulates the number of axonal branches via Bax/Bak in the axons of corticospinal neurons. Early postnatal inactivation of Bax/Bak in motor cortex results in increased axonal branches in the spine of these animals when they reach adulthood. As a consequence, connectivity’s fine-tuning is lost and animals fail to acquire fine voluntary movements (Gu et al., 2017).

Normal brain functions depend on proper synaptic activity. Long-term potentiation (LTP) and long-term depression (LTD) are long-lasting modifications of synapses in the hippocampus region. Activation of caspase-3 during LTD occurs without cell death (Li et al., 2010; Simon et al., 2012) and facilitates the internationalization of AMPA receptor in the postsynaptic membrane. Furthermore, caspase-3 promotes modifications in AMPA-type receptor that lead to alterations of glutamatergic synaptic transmission and plasticity in Tg2576-APPSwe mice. Notably, pharmacological inhibition of caspase-3 activity in these mice rescued the observed AD-like phenotypes (D’Amelio et al., 2011).

### Caspases and Neurotoxic Protein Aggregates

Caspases play a significant role in the pathogenesis of different neurodegenerative diseases including AD, PD and Huntington’s disease (HD), since they are able to modify the properties of different neurotoxic proteins aggregates (amyloid precursor protein (APP), Tau, α-synuclein (α-Syn) and huntingtin (htt) through cleavage.

Under apoptotic conditions, caspases cleave APP, either directly or following γ-secretase generation of the AICD fragment, to generate the C31 and Jcasp peptides [for more information see review (Nhan et al., 2015)]. While AICD and C31 are associated with cell death *in vitro* (Lu et al., 2000; Bertrand et al., 2001; Park et al., 2009), the impact of this *in vivo* may be minimal, since mice bearing mutant caspase-resistant APP displayed no rescue in learning or memory in a mouse model of AZ (Harris et al., 2010).

Tau is another substrate processed by caspases early in the progression of AD (Rissman et al., 2004). Cleaved Tau promotes nucleation-dependent filament formation which is phosphorylated by glycogen synthase kinase-3b in a process mediated by caspase-3 (Chu et al., 2017). Recently, it has been shown that caspase-2 specific cleavage in Tau provokes an inadequate sorting of Tau toward the dendritic spines, promoting cognitive impairment and synaptic dysfunction (Zhao et al., 2016). Interestingly, cleavage of APP promotes phosphorylation of Tau in different residues, in a process where C31 binds to the asparagine, proline, threonine, tyrosine (NPTY) motif of the catalytic subunit of the phosphatase PP2A, repressing its activity (Park et al., 2012).

α-Syn is an abundantly expressed neuronal protein localized in the presynaptic terminals of neurons. α-Syn is key to understanding the etiology of a group of overlapping neurodegenerative disorders called α-synucleinopathies, including PD. α-Syn is processed through caspase-1 forming a truncated protein whose effect is to accelerate the formation of aggregates as compared to the full-length form of α-Syn (Wang et al., 2016). Interestingly, truncated α-Syn itself stimulates caspase-1 activation which promotes more cleavage of α-Syn, in a positive feedback loop (Ma et al., 2018).

Finally, caspase cleavage of htt induces it’s accumulation in the nuclei of neurons of HD patients (Graham et al., 2010). Caspase-6 cleaves htt, and caspase-6 activation has been observed before the onset of motor abnormalities both in mouse and human HD brains (Graham et al., 2010). Strikingly, *in vivo* experiments using YAC mice expressing caspase-6-resistant cleavage of htt, showed no striatal neurodegeneration. Recently a new caspase cleavage site of htt has been described, in this case depending on caspase-1 activity that promotes aggregation of mutant htt (Martin et al., 2019).

While the evidence gathered in PD and HD studies suggests that caspase-dependent processing of α-Syn and htt may play a role in the pathogenesis of the diseases, conflicting results for APP *in vitro* and *in vivo* have been observed in AD models, requiring further investigation.

Also, the experiments to study cleavage of APP by caspases have been performed under apoptotic conditions, which makes difficult to conclude if the caspase cleavage of APP plays any pathological role or is just “collateral damage” that occurs during apoptosis. Further experiments under non-cell death conditions should be performed to resolve this issue.

### Caspases and Autophagy and Mitochondria Biogenesis

Autophagy is a catabolic process that delivers cytoplasmic constituents into lysosome for degradation and eventual recycling. Under physiological conditions, autophagy promotes cell survival by elimination of damaged organelles and proteins aggregates (Zare-Shahabadi et al., 2015). However, autophagy has also been linked to cell death, either promoting it (Das et al., 2012) or at least being associated with it (Kroemer and Levine, 2008). Evidence suggests that autophagy may be controlled by caspases during apoptosis (Tsapras and Nezis, 2017). However, evidence of the interaction between autophagy-related proteins and caspases in the CNS are still scarce. In primary cultures of cortical neurons treated with rotenone, lack of caspase-2 prolonged cell survival by enhancing autophagy. However, the cells die eventually via necrosis (Tiwari et al., 2011).

A recent study showed that caspase-3-induced Beclin-1 cleavage and subcellular redistribution of the Beclin-1 N-terminal into the nucleus has been shown in neuron-glia co-cultures with Aβ_1__–__42_ and APPSwe/PS1dE9 mice. The authors hypothesized that cleavage of Beclin-1 by caspase-3 could affect autophagy and lead to defective protein clearance and neuronal death (Wang et al., 2017).

Despite all these roles that we have discussed in this mini-review, new and exciting roles are still emerging for caspases in the CNS. For instance, in a recent study performed by Kim et al. (2018) in dopaminergic neurons, the authors described how procaspase-3 acts as a regulator for mitochondria biogenesis without affecting autophagy. They showed that TFAM, Nrf-1, and PGC-1a (transcriptional activators of mitochondrial biogenesis) are regulated by procaspase-3. Furthermore, in the same study, the authors show that lack of procaspase-3 in dopaminergic neurons dramatically reduced electron transport chain complex I, II, and IV activity. Interestingly, treatment with caspase-3 inhibitor failed to mimic the observed effects thus raising the view that caspase-3-dependent mitochondrial biogenesis is independent of its catalytic activity.

## Discussion

Within the last decade, there has been an exponential growth in the number of studies of non-apoptotic functions of caspases. Some of these non-apoptotic roles have been extensively studied in specific cell types, such as the inflammasome in immune cells (Martinon et al., 2002). In the CNS, while many of these non-apoptotic functions have been studied in microglia cells, few reports have been published for neurons, astrocytes and ODCs in comparison. Here we attempt to shed light onto some “old and new” non-apoptotic functions for caspases in these cell types, which have been gathering momentum in recent years and their relevance under pathological conditions.

Why does the activation of executioner caspases not translate always into an apoptotic process? A possible explanation could be sequestration of effector caspases into different subcellular compartments (Li et al., 2010; Kavanagh et al., 2014; Amcheslavsky et al., 2018) and/or cleavage of non-cell death related substrates (Acarin et al., 2007).

Different manners in which caspases affect cellular signaling continue to emerge. Besides the diverse mechanisms employed to activate different caspases already commented in this mini-review, it has been shown that the reduction of the basal activity of caspase-3 promotes a change toward a tumor supportive phenotype in microglia cells in contact with glioma cells (Shen et al., 2016). Also, and in agreement with this, in embryonic stem cells (ESCs), basal caspase-3 activity regulates cell differentiation of these cells through Nanog processing (Baena-Lopez et al., 2018). We have also discussed previously in this mini-review how the zymogen (procaspase-3) mediates the mitochondrial biogenesis independently of its enzymatic activity (Kim et al., 2018). It is possible that these functions of caspase-3 can be extended to other caspases, opening new possibilities in the field of caspase biology, for new mechanistic roles of caspases with relevance not only limited to the CNS but applicable to all cell types.

## Author Contributions

MB and AE-O conceived the main outline. MB wrote the manuscript. All authors searched the references and decided the contents of the mini review.

## Conflict of Interest

The authors declare that the research was conducted in the absence of any commercial or financial relationships that could be construed as a potential conflict of interest.
